# Supplementary material for: A linear reciprocal relationship between robustness and plasticity in homeostatic biological networks
Source: PLoS One. 2023 Jan 26;18(1):e0277181. doi: 10.1371/journal.pone.0277181 (PMC9879506; doi:10.1371/journal.pone.0277181)
Supplement: Attachment — Submitted filename: response.pdf [file pone.0277181.s004.pdf]

Below we address the corrections suggested by the reviewers in a point-by-point manner. Note that the reviewers' comments are noted with brackets, and the corrections are highlighted by red in the revised manuscript.

Reviewer #1:

[*This is the revision of a manuscript I reviewed a few months ago (PONE-D-22-14450). The scientific content of the manuscript was sound, and in spite of a rather technical content, I found it suitable for publication in PLoS ONE. I had a major concern and a few comments, each having being addressed in the response to the reviewers, and in the text of the paper. The most significant changes were in the abstract (which as mostly rewritten), in the methods (many of the reviewers' comments were related to the methods), and in the results/discussion, with additional explanations provided in 6 paragraphs, as well as an additional figure (new figure 5). This revision is substantially longer than the original version (~ 2 pages), and has the same pros (scientifically sound) and cons (probably accessible to specialists only) than the first submission. Most of the changes in the manuscript are verbatim copies of the responses to the reviewers, which is not problematic per se (although it does not help to make the manuscript more accessible). Note that data/software sharing is minimal, and (in my opinion) does not match at all PLOS transparency guidelines.*]

Thank you for your comments.
As you pointed out, it is harder for non-specialist to access our revised manuscript than the first submitted version. Thus, we moved some sentences to the reference list (as a footnote) to improve the readability.

[*About my comment #1 (what the optimal gene expression fixed at the beginning of the simulations), the authors now make it clear how the optimal expression was determined. Although I may not necessarily agree with the modeling choice (which implies that the network is completely shut down when the environmental signal is at zero), the authors now discuss the consequences of this model setting, and cite the paper by Siegal & Bergman about the relationship between stabilizing selection for an optimum and selection for network stability, which is probably more than enough to inform the reader about potential pitfalls.*]

Thank you for your positive evaluation.

[*About my comment #2 (not enough details to reproduce the simulations): the authors have expanded the model description, and it is probably easier now to re-implement the model. Note that the author's statement about data availability states that "data are fully available without restriction", which I find misleading: the authors have provided only summary statistics (basically, the location of the points on the figures). As far as I can tell, there is nothing available about the simulation code (the implementation of the model), nothing about the analysis code (how data was extracted from the simulation output, how summary statistics were computed from the 100 replicates, etc). This is as far to "open science" as possible, and, as far as I can tell, contradicts directly PLoS One policies about data sharing (https://journals.plos.org/plosone/s/data-availability) and software sharing (https://journals.plos.org/plosone/s/materials-software-and-code-sharing).*]

Since the total number of files is huge (100 files for each point in each figure), we uploaded the averaged data as samples in the last time. In the revised manuscript, we uploaded the codes for Fig. 2AB, for Figs. 2CD, 4, and 5, for Fig. 3ABCD, for Fig. 3E, and for Fig, 3F, to assure the reproducibility.

[*My comment #3 (structure of the manuscript) has been fully addressed.*

My comment #4 has been addressed (in the caption of the figure, not in the figure itself, not a big deal).

My comment #5 (range of evolution for DeltaX) is now addressed in the manuscript.

My comment #6 has been addressed in a very satisfactory way, with a new figure and more insightful results.]

Thank you for your positive comments.

[*In the text: line 91, "We set the asexual reproduction" sounds awkward (perhaps "Reproduction was asexual"?).*]

We have corrected the sentence.
